# Bacterial co-cultivation for the degradation of polystyrene plastics

**DOI:** 10.1016/j.engmic.2025.100232

**Published:** 2025-08-19

**Authors:** Yingbo Yuan, Tianyuan Su, Yi Zheng, Baoyue Liu, Yuanfei Han, Zhongcan Wang, Quanfeng Liang, Longyang Dian, Qingsheng Qi

**Affiliations:** State Key Laboratory of Microbial Technology, Shandong University, Qingdao 266237, China

**Keywords:** Polystyrene, Biodegradation, Environmental pollution, Plastic package, Co-cultivation

## Abstract

Polystyrene (PS) is a polyolefin plastic that is used extensively in food packaging. The chemical structure of PS is extremely stable owing to its C-C backbone and styrene rings, making it highly resistant to biodegradation, which causes serious environmental pollution and health threats. Although certain microorganisms have been reported to degrade PS waste, most studies have focused on the changes in the molecular weight and surface structure of plastics. These slight degradation phenomena make it extremely difficult to detect the degradation products, thus challenging the definitive demonstration of PS degradation. This study investigated the co-cultivation of the polyolefin plastic-degrading bacterium *Raoultella* sp. DY2415 and the benzoic acid bioconversion strain *Pseudomonas putida* KT2440-ΔRBC. BA is a possible degradation product of PS and can be converted by *P. putida* KT2440-ΔRBC into the high value-added compound muconic acid (MA). After co-cultivation, MA was detected in the medium, indicating that *Raoultella* sp. DY2415 degraded PS and generated BA, which was subsequently utilized by *P. putida* KT2440-ΔRBC for MA synthesis. This study demonstrated the biodegradation of PS and the synthesis of MA through a fully biological process, thereby promoting the circular economy of plastics.

## Highlights

• Microbial co-culture was proposed for the management of polystyrene waste.
• The degradation of PS by co-culture was 2.85 times greater than pure culture.
• Polystyrene was recycled to muconic acid through a fully biological process.
• Microbial co-culture exhibited the feasibility of PS biodegradation and upcycling.

## Graphical abstract

**Figure:**
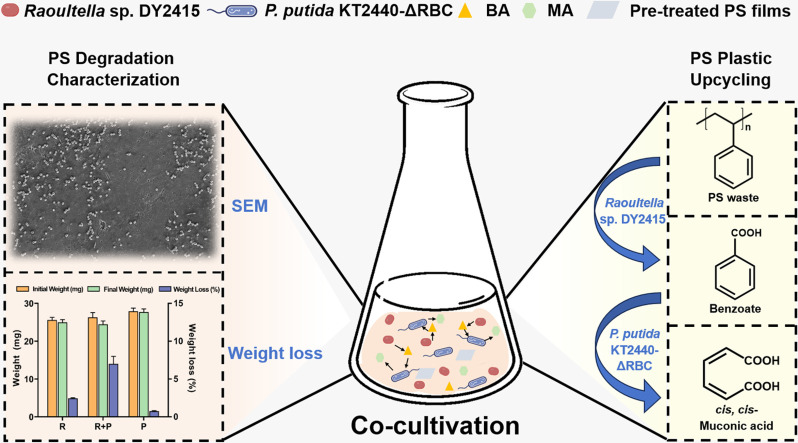

## 1 Introduction

Polystyrene (PS) is a common petroleum-based thermoplastic and is the fifth most produced plastic globally [1,2]. PS is widely used in food packaging, such as vegetable cling films, disposable food containers, and foam insulation boxes [3]. In China, the annual production of disposable plastic food containers reached a staggering 45 billion units [4]. Moreover, PS has a stable chemical structure and is difficult to biodegrade, thus generating large amounts of plastic waste and microplastics in the environment [5]. Studies have reported that microplastics can enter the human body via inhalation and ingestion, posing potential health threats [6,7]. Moreover, traditional plastic waste disposal methods include landfilling and incineration, resulting in secondary pollution of the soil and air [8].

The biodegradation of PS has attracted widespread attention [[9], [10], [11]]. Some microorganisms with the potential to biodegrade PS have also been reported. *Bacillus cereus* (OR268710) isolated from heavily polluted coastal areas exhibited degradation activity resulting in 14.13 ± 2.41% weight loss of PS within 42 days of cultivation, with scanning electron microscopy (SEM) showing surface erosion with cracks [12]. Similarly, *Acinetobacter* sp. AnTc-1, isolated from the larvae of *Tribolium castaneum*, was reported to degrade PS and exhibited a 13% average molecular weight reduction after 60 days of incubation [13]. Because of its high molecular weight and strong hydrophobicity, PS is highly resistant to biodegradation [14]. Indicators that demonstrate the degradation of PS plastics mainly include weight loss, changes in physical and chemical properties, and average molecular weight distribution [15]. However, some studies suggest that changes in the surface structure and slight weight reduction are not direct indications of PS biodegradation [16]. Therefore, an increasing number of studies have attempted to provide direct evidence of the presence of degradation products using methods such as gas chromatography-mass spectrometry (GC-MS) and high-performance liquid chromatography-mass spectrometry (HPLC-MS) [17]. Currently, only a few studies have reported identifying the degradation products of PS, with only trace quantities of metabolic intermediates being detected. *Pseudomonas putida* strain H-01, which is reported to degrade PS, can only detect a few monomeric substances, including styrene, toluene, xylene, and other small-molecule compounds [18]. *Microbacterium esteraromaticum* SW3 isolated from the soil has been reported to degrade PS and produce a small number of alkanes, ketones, and other degradation products [19].

Co-cultivation systems are important for the degradation of pollutants such as polycyclic aromatic hydrocarbons (PAHs), phenanthrene, and plastic waste [20,21]. In recent years, co-cultivation has been used for upcycling plastic waste [22,23]. For example, polyethylene terephthalate (PET) degradation was coupled with polyhydroxybutyrate (PHB) production through the co-cultivation of *Yarrowia lipolytica* Po1f and *Pseudomonas stutzeri* strains [24]. To further upcycle PET waste, PET oligomers were degraded and used to synthesize polyhydroxyalkanoates (PHA) by co-cultivating engineered *E. coli* BL21 (DE3) and *P. putida* KT2440, providing a biological strategy for the upcycling of PET oligomers and promoting the plastic economy [25]. Furthermore, co-cultivation of microorganisms can lead to significant plastic biodegradation, such as weight loss and improved degradation rates [26]. For instance, a co-cultivation system comprising the bacteria SUST B_1_, SUST B_2_, and SUST B_3_ has been reported to improve the degradation rate of poly (butylene adipate-co-terephthalate) (PBAT) compared to that of their respective pure isolates [20]. Similarly, a co-culture system containing *Rhodococcus* sp. WB9 and *Mycobacterium* spp. WY10 was constructed to efficiently degrade phenanthrene at a higher degradation rate than that of their respective monocultures [27].

In a previous study, a *Raoultella* spp. DY2415 strain was isolated from petroleum-contaminated soil in Shandong, China [28]. Through SEM, Fourier transform infrared spectrometry (FTIR), and weight loss measurements, we verified the typical degradation of polyethylene (PE) and PS films by *Raoultella* sp. DY2415, whereas degradation products were not detected. Therefore, to further verify PS degradation, we developed a co-cultivation system that included *Raoultella* sp. DY2415, a bacterium with potential PS-degrading properties, and *P. putida* KT2440-ΔRBC, a bacterium capable of converting the PS plastic degradation product benzoate (BA) into the high value-added compound muconic acid (MA). The co-cultivation system more effectively degraded PS than pure *Raoultella* sp. DY2415. Furthermore, we achieved the direct conversion of PS plastic into a high value-added chemical, MA, through a co-cultivation system, providing a new method for upcycling PS waste.

## 2 Materials and methods

### 2.1 Strains and bacterial cultivation

*Raoultella* sp. DY2415 was cultivated at 37°C on a rotary shaker (220 rpm) in Luria–Bertani (LB) medium (5 g/L yeast extract, 10 g/L peptone, and 10 g/L NaCl). *P. putida* KT2440 and derived strains were conventionally cultivated at 30°C on a rotary shaker (220 rpm) in LB medium. The strains used in this study are listed in Table S1. The PS films were then exposed to a UVB lamp (λ = 313 nm) for 20 d. Then, the pre-treated PS films were washed with 75% ethanol and dried on a clean laminar flow bench. Finally, the pre-treated PS films were weighed and added to the medium as substrates. After incubation, the pre-treated PS films were collected, washed with 2% sodium dodecyl sulfate (SDS), 75% ethanol, and sterilized water, and then weighed to calculate the weight loss.

### 2.2 Constructions of plasmids and strains

*cat*RBC gene insertion and deletion were performed via two-step recombination using the vector pK18mobsacB to obtain the MA-accumulating engineered bacterium *P. putida* KT2440-ΔRBC [29]. The plasmids used in this study are listed in Supplementary Table S1. The sequences of the primers used are listed in Table S2. Initial recombination into the chromosome was selected based on kanamycin resistance in LB plates containing 25 mg/L kanamycin, and the second recombination was selected based on the sucrose lethal gene (*sac*B) in LB plates containing 20% sucrose [25]. Engineered bacteria were tested in LB medium supplemented with 20 mM BA as the substrate, and the control was cultured in LB without BA. Samples were collected every 24 h and analyzed using HPLC after centrifugation and filtration. Each group was set up in triplicate.

### 2.3 Co-cultivation of *Raoultella* sp. DY2415 and *P. putida* KT2440-ΔRBC

*Raoultella* sp. DY2415 and *P. putida* KT2440-ΔRBC were cultivated in LB medium at 30 °C on a rotary shaker (220 rpm) for one week. The medium was supplemented with pre-treated PS films that were weighed beforehand. Single cultures of *Raoultella* spp. DY2415 and *P. putida* KT2440-ΔRBC were used as the controls. The initial OD_600_ was the same for the individual cultivation of *Raoultella* sp. DY2415, individual cultivation of *P. putida* KT2440-ΔRBC, and co-cultivation of *Raoultella* sp. DY2415 and *P. putida* KT2440-ΔRBC. Three parallel experiments were performed for each sample. Samples were collected every 2 days.

### 2.4 Optimization of co-cultivation conditions

Two co-culture temperatures were tested, 30 °C and 37 °C. *Raoultella* sp. DY2415 was inoculated at 37 °C from overnight cultures into 50 mL LB medium, and *P. putida* KT2440-ΔRBC was inoculated at 30 °C from overnight cultures into the same 50 mL LB medium under identical inoculation conditions (*Raoultella* sp. DY2415: *P. putida* KT2440-ΔRBC = 1:1).

### 2.5 BA and MA detection

The cultures were centrifuged at 12,000 rpm for 5 minutes. The supernatants were then filtered with a 0.22 µm filter membrane and analyzed via HPLC. Products in the culture broth, including BA and MA, were detected using a photodiode array detector at 245 nm in a SHIMADZU LC-20A HPLC system equipped with a Discovery HS C18 (4.6 × 250 mm) column [29]. Chromatographic separation employs isocratic elution with a specified mobile phase. The mobile phase consisted of 0.1% (v/v) trifluoroacetic acid and 100% (v/v) acetonitrile. The flow rate and column temperature were set to 0.8 mL/min and 40 °C, respectively. The elution time was 8 min.

### 2.6 Weight loss analysis

After incubation, the pre-treated PS films were collected and washed with a 2% SDS buffer, 75% ethanol, and sterilized water [28]. After air drying, the films were weighed using an analytical balance (METTLER TOLEDO AB104S) with a resolution of 0.1 mg. Weight loss (%) was calculated using the formulae below [30]:

Weightloss(%)=[(Iw−Fw)÷Iw]×100

where *Iw* is the initial weight and *Fw* is the final weight.

### 2.7 Scanning electron microscopy (SEM) analysis

For SEM analyses, the plastic films were soaked in 2.5% glutaraldehyde for 3 h at 4°C and dehydrated with 30–100% graded ethanol for 15 min, then critical-point-dried with CO_2_ using an EM CPD300 Critical Point Dryer (Leica, Germany). The dried specimens were sputter-coated for 4 min with gold and platinum (10 nm) to increase the surface conductivity for analysis using a Quanta 250 FEG SEM (FEI, USA) operating at an accelerating voltage of 5 kV [28].

### 2.8 High-performance liquid chromatography-mass spectrometry (HPLC-MS) analysis

HPLC-MS analysis of products released from the pre-treated PS films was performed on a High Resolution Q-TOF mass spectrometry (impactHD, Germany) system equipped with a Discovery HS C18 (4.6 × 250 mm) column. Mobile phase A was 0.1% formic acid, mobile phase B was acetonitrile, the flow rate was 0.8 mL/min, and the effluent was monitored at a wavelength of 245 nm. ESI was used as the ionization method. The capillary voltage was set as 4000 V, the nebulizer at 0.4 bar, the dry gas flow rate was 4.0 L/min, and the dry temperature was 200 °C. The molecular weight collection range was 50–700 m/z.

### 2.9 Statistical analysis

Experimental data were collected from at least three independent experiments, with error bars representing the standard deviations. Statistical analysis was conducted using Student’s *t*-test, where the asterisk (*) denotes the level of statistical significance (0.05<ns<1, *p<0.05, **p<0.01, ***p<0.001, ****p<0.0001). *P*-values below 0.05 were considered statistically significant [31].

## 3 Results and discussion

3.1 Biodegradation of pre-treated PS films by *Raoultella* sp. DY2415

**Fig. 1:**
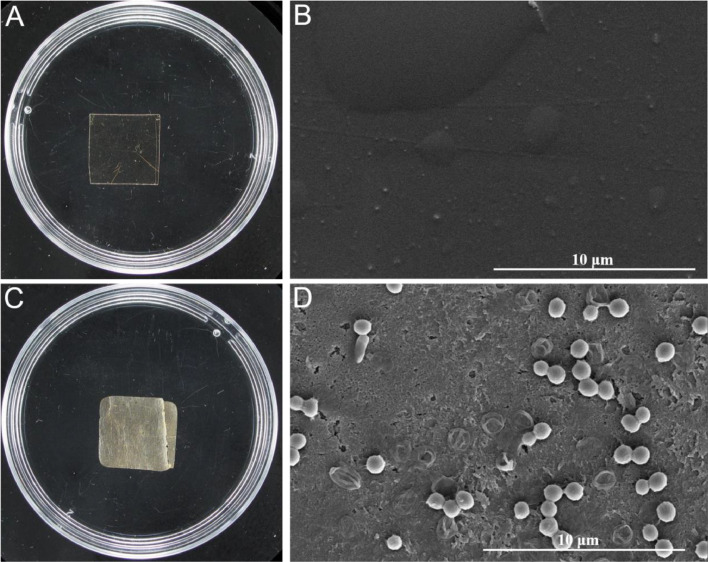
The *Raoultella* sp. DY2415 strain degrades pre-treated PS films. Morphology of the pre-treated PS films before (A) and after (C) incubation with *Raoultella* sp. DY2415 for one week. Scanning electron microscopy observation of the pre-treated PS films before (B) and after (D) incubation with *Raoultella* sp. DY2415 for one week.

**Fig. 2:**
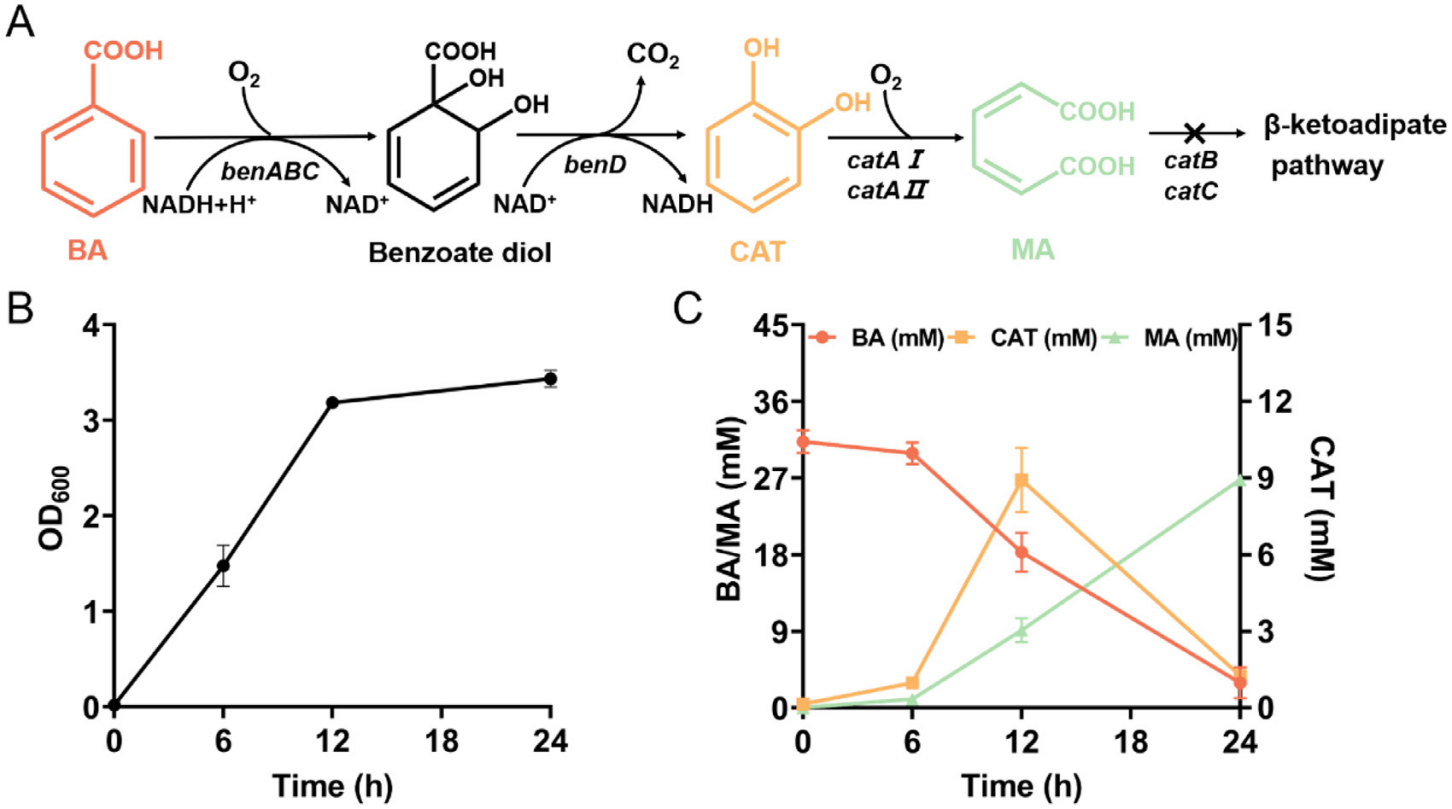
Engineering *P. putida* KT2440-ΔRBC to metabolize benzoate (BA) and generate muconic acid (MA). The metabolic pathway of BA in *P. putida* KT2440 (A). The growth curve of *P. putida* KT2440-ΔRBC in Luria–Bertani (LB) medium supplemented with BA (B). Bioconversion of BA to produce MA by *P. putida* KT2440-ΔRBC (C). *P. putida* KT2440-ΔRBC was cultivated in LB medium supplemented with 25 mM BA at 30°C, 220 rpm. Three parallel experiments were carried out for each sample.

*Raoultella* sp. DY2415 is a promising polyolefin plastic-degrading strain that was isolated from petroleum-contaminated soil [29]. To verify that PS was indeed degraded by *Raoultella* sp. DY2415, we used pre-treated PS films that were UV-irradiated for 20 d as the substrate for the biodegradation experiment. After one week of incubation, we observed that the corners of the pre-treated PS films incubated with *Raoultella* sp. DY2415 became rounded from their original sharp edges (Fig. 1A and 1C). In addition, the surface of the pre-treated PS film was yellow and rough. In our previous study, a PS film without UV pre-treatment was incubated with *Raoultella* sp. DY2415 showed a 2% weight loss during the first week of incubation [28]. In this study, we found that the weight of the pre-treated PS film decreased from 18.1 mg to 17.6 mg, with a weight loss of 2.8%, indicating that UV pre-treatment can accelerate the biodegradation of PS by *Raoultella* sp. DY2415. Furthermore, SEM observations revealed that the pre-treated PS film exhibited bulging on its surface (Fig. 2B), which may have facilitated microbial adhesion. As illustrated in Fig. 1D, some bacteria attached to the pre-treated PS film generated biofilm-like substances, which were considered to play an important role in PS degradation. Compared to the SEM results for *Raoultella* sp. DY2415-degraded PS films without UV pre-treatment, the pre-treated PS films became rough and generated many pores. The hydrophobicity of the pre-treated PS films was reduced owing to surface oxidation caused by UV aging. Therefore, the bacterial community and enzymes secreted by the microorganisms can easily adhere to the polymer [32], promoting the decomposition of plastics into smaller monomers through oxidation and depolymerization. Notably, holes were generated on the surfaces of the pre-treated PS films (Fig. 1D). These results indicate that *Raoultella* sp. DY2415 may degrade pre-treated PS films.

PS polymers primarily consist of a carbon chain and aromatic ring side chains, and their degradation can be characterized by main-chain (C-C) or side chain (benzene ring) oxidation and aromatic compound cleavage [33]. Previous studies have proposed potential metabolic routes for PS; through a series of enzymatic reactions, PS is converted to aromatic residues, such as styrene, benzoylformate, BA, and other benzene ring compounds [10,34]. However, we did not detect any PS degradation products in *Raoultella* sp. DY2415.

As a common aromatic thermoplastic, PS may produce small-molecule degradation products with benzene rings, such as benzoic acid [35,36]. In a previous study, Kyoto Encyclopedia of Genes and Genomes metabolic pathway analysis indicated that *Raoultella* sp. DY2415 (Bioproject accession: PRJNA898983) encodes 36 genes involved in BA metabolism (Table S3). It included the toluate 1,2-dioxygenase electron transfer component, 1,6-dihydroxycyclohexa-2,4-diene-1-carboxylate dehydrogenase, and catechol 1,2-dioxygenase, which are involved in the BA metabolism pathway of *P. putida* KT2440. However, *Raoultella* sp. DY2415 also possesses distinct benzoic acid metabolism genes absent from *P. putida* KT2440, including protocatechuate 3,4-dioxygenase beta chain, protocatechuate 3,4-dioxygenase alpha chain, 4-hydroxy-2-oxovalerate aldolase, and several hypothetical proteins of unknown functions. BA is a small molecule that is metabolized by many microorganisms. We incubated *Raoultella* sp. DY2415 with LB medium supplemented with BA and found that the concentration of BA in the *Raoultella* sp. DY2415-containing medium decreased over the incubation period (Fig. S1). Therefore, we speculate that *Raoultella* sp. DY2415 degraded the pre-treated PS films, and the resulting degradation product, BA, may have been directly metabolized.

### 3.2 Conversion of BA to MA by engineering P. putida KT2440

*Raoultella* sp. DY2415 is a wild-type strain isolated from petroleum-contaminated soil that cannot be genetically engineered [28]. Therefore, to identify the degradation products and definitively confirm the biodegradation of the PS film by *Raoultella* sp. DY2415, a bacterial species capable of metabolizing BA and producing a transparent substance was identified. *P. putida* is a promising bacterium that can manage industry-derived synthetic waste, including plastics, oils, and agricultural waste, into high-value products, such as platform chemicals (e.g., *cis, cis*-muconic acid, and adipic acid) and biopolymers (e.g., polyhydroxyalkanoates) [37]. Furthermore, *P. putida* KT2440 has native BA metabolism [38]. First, BA is converted to BA diol by BA 1,2-dioxygenase (EC 1.14.12.10), which is encoded by *benABC*. The oxidative decarboxylation of BA diol to catechol is performed by BA diol dehydrogenase (EC 1.3.1.25), which is encoded by *benD*. Ring fission of catechol between hydroxyl groups is catalyzed by CatA, encoded by *catA*, to form MA; the latter metabolite is then converted into muconolactone by muconate cycloisomerase (EC 5.5.1.1), which is encoded by the *catB* gene. Finally, muconolactone is converted into tricarboxylic acid cycle intermediates after several metabolic steps (Fig. 2A).

MA is an important unsaturated dicarboxylic acid that can undergo various reactions as a building block or intermediate to produce commodities and specialty chemicals, including commercially important bulk chemicals such as adipic acid, terephthalic acid, and trimellitic acid [39,40]. These chemicals have a wide variety of applications in the manufacture of nylon-6,6, polytrimethylene terephthalate, plasticizers, and engineering polymers [41]. BA is one of the most common raw materials for MA production because of its stability, water solubility, and low price [41]. Therefore, we engineered *P. putida* KT2440 for co-cultivation with *Raoultella* spp. DY2415, and MA was selected as the final detection product.

To accumulate MA, we knocked out catechol metabolism and the transcriptional regulator gene *catRBC* in *P. putida* KT2440 to disrupt the metabolic conversion of MA to muconolactone (Fig. S2) to obtain an MA-accumulating strain. We found that *P. putida* KT2440-ΔRBC can generate 27 mM MA in 24 h with 30 mM BA as the substrate (Fig. 2). Additionally, it simultaneously accumulated intermediate catechol products.

In addition, we also set the negative control, incubating the engineered bacteria *P. putida* KT2440-ΔRBC in LB medium without BA. We found that *P. putida* KT2440-ΔRBC cannot generate MA when cultivated in LB medium without BA. Therefore, we can utilize the engineered bacteria *P. putida* KT2440-ΔRBC to detect trace amounts of BA in the incubation medium. When MA was detected in the co-culture medium, *Raoultella* sp. DY2415 biodegraded the pre-treated PS film and produced BA, which was simultaneously utilized by *P. putida* KT2440-ΔRBC for MA synthesis.

### 3.3 Co-cultivation of Raoultella sp. DY2415 and P. putida KT2440-ΔRBC

**Fig. 3:**
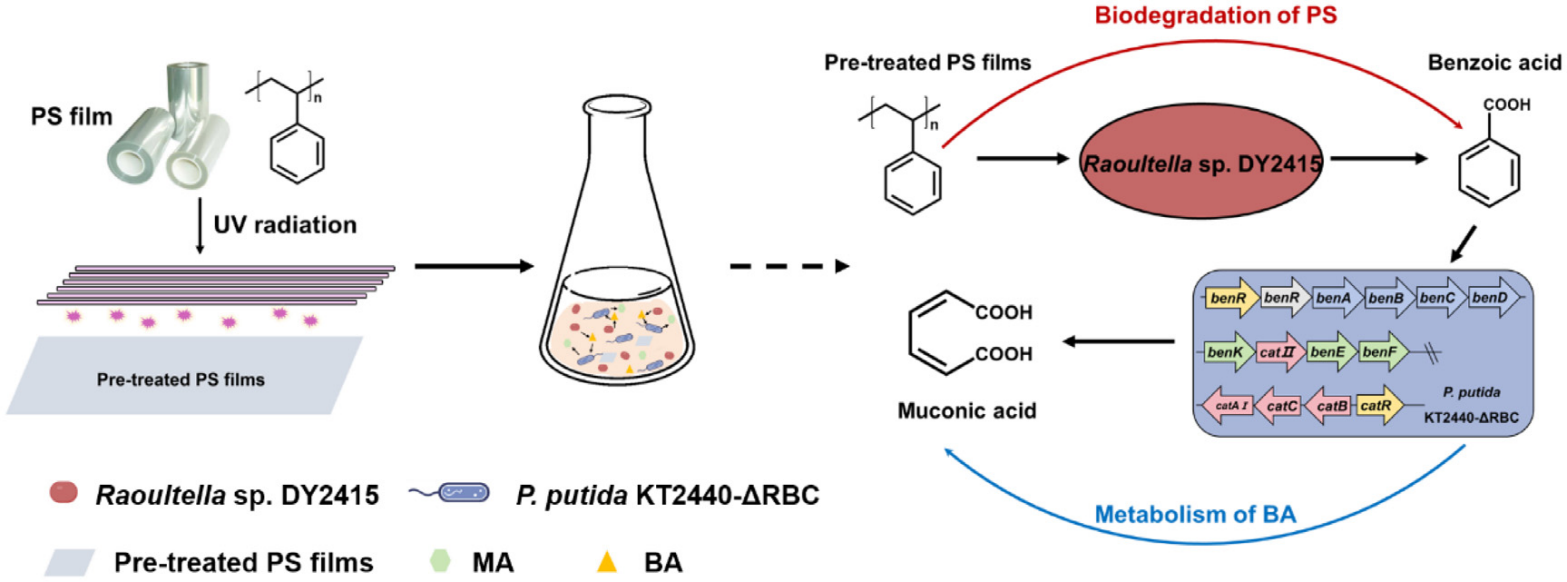
Scheme of *Raoultella* sp. DY2415 and *P. putida* KT2440-ΔRBC co-cultivation to biodegrade polystyrene and synthesize muconic acid.

After constructing the engineered bacterium *P. putida* KT2440-ΔRBC, we proposed a co-cultivation model using *Raoultella* sp. DY2415 and *P. putida* KT2440-ΔRBC, which can synthesize the high-value compound MA and simultaneously demonstrate the PS degradation by *Raoultella* sp. DY2415 (Fig. 3).

**Fig. 4:**
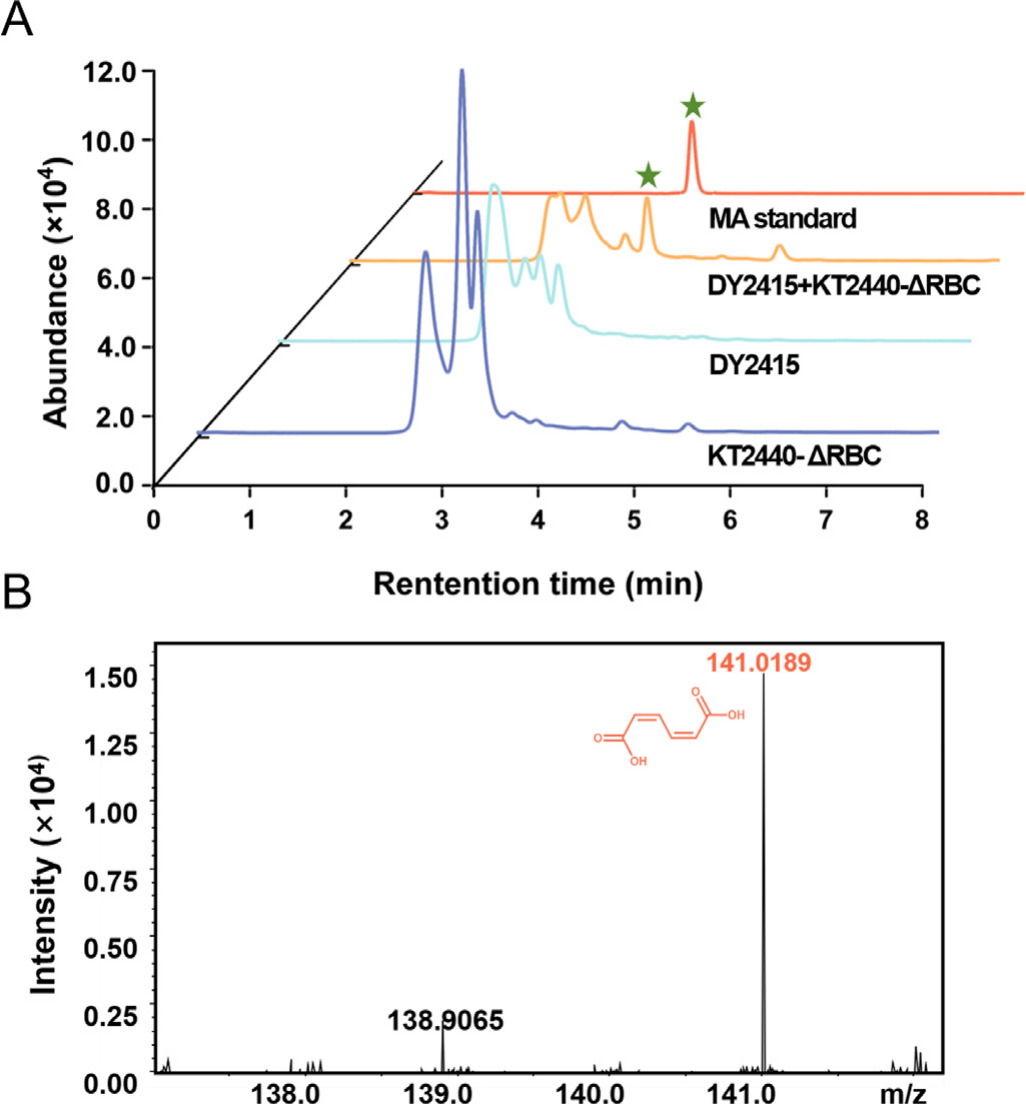
Co-cultivation system degraded polystyrene (PS) films and synthesized muconic acid (MA). HPLC results of *Raoultella* sp. DY2415 and *P. putida* KT2440-ΔRBC co-cultivation, *Raoultella* sp. DY2415 individual cultivation, and *P. putida* KT2440-ΔRBC individual cultivation with pre-treated PS film for one week (A). HPLC-MS result of *Raoultella* sp. DY2415 and *P. putida* KT2440-ΔRBC co-cultivation using pre-treated PS film as the substrate for one week (B).

Next, we attempted to co-culture *Raoultella* sp. DY2415 and *P. putida* KT2440-ΔRBC. The two negative control groups (individual *Raoultella* sp. DY2415 and individual *P. putida* KT2440-ΔRBC) and one blank control group (LB medium supplemented with pre-treated PS films, without bacterial inoculation) were set up under the same cultivation conditions. After one week of incubation, the supernatant composition was analyzed using HPLC. MA was only detected in the co-cultivation group; the three control groups did not show any MA absorption peaks (Fig. 4). To further confirm that the absorption peak in the co-culture supernatant was indeed MA, we performed HPLC-MS (Fig. 4B and Fig. S3). The mass spectrum was consistent with that of the MA standard sample, demonstrating the presence of MA in the co-culture supernatant (Fig. 4B and S3). These results confirmed our hypothesis that *Raoultella* sp. DY2415 can biodegrade the pre-treated PS films and generate BA, which is then utilized by the engineered bacterium *P. putida* KT2440-ΔRBC to generate MA. However, it has been reported that under prolonged UV aging, many small-molecule organic intermediates, such as BA and phenol, can gradually form on the surface of PS [36,42]. To rule out the possibility that BA was generated by UV irradiation, we cultured *P. putida* KT2440-ΔRBC only in the medium supplemented with the pre-treated PS film and did not detect MA. This suggests that MA was synthesized from BA produced from PS degradation by *Raoultella* sp. DY2415. In addition, the MA product was not detected in the LB medium supplied with pre-treated PS films, ruling out the influence of the medium on its synthesis.

We also optimized the incubation conditions for co-cultivation. The optimal growth temperature for *Raoultella* sp. DY2415 is 37 °C [28], whereas that for *P. putida* KT2440 is 30 °C [38]. Therefore, we tried to co-culture these two microorganisms at 30°C and 37°C, respectively. The results showed no accumulation of MA at 37°C, whereas MA was detected after 12 h at 30°C (Fig. S4). Therefore, we chose 30 °C as the optimal co-cultivation temperature.

The inoculation proportion of each microorganism in the co-culture also had a significant effect on strain growth. Singh et al. set different inoculation ratios for the co-cultivation of *Diplosphaera mucosa* and *Scenedesmus obliquus* and found that the highest biomass productivity was obtained at a co-cultivation ratio of 1:4 [43]. Therefore, we investigated the effect of different inoculation ratios of the two investigated bacteria on MA production. Three different inoculation ratios of *Raoultella* sp. DY2415 and *P. putida* KT2440-ΔRBC were then investigated: 1:1, 3:7, and 7:3 (Fig. S5). The inoculation ratio affected MA accumulation. Similar to the previous results, MA production decreased when the proportion of *Raoultella* sp. DY2415 exceeded that of *P. putida* KT2440-ΔRBC, indicating that a balanced consortium ratio is required for efficient PS degradation and conversion (Fig. S5). The optimal ratio for co-cultivation of *Raoultella* sp. DY2415 and *P. putida* KT2440-ΔRBC that could generate the most MA is 1:1 (Fig. S5).

### 3.4 Characterization of PS biodegradation during co-cultivation

**Fig. 5:**
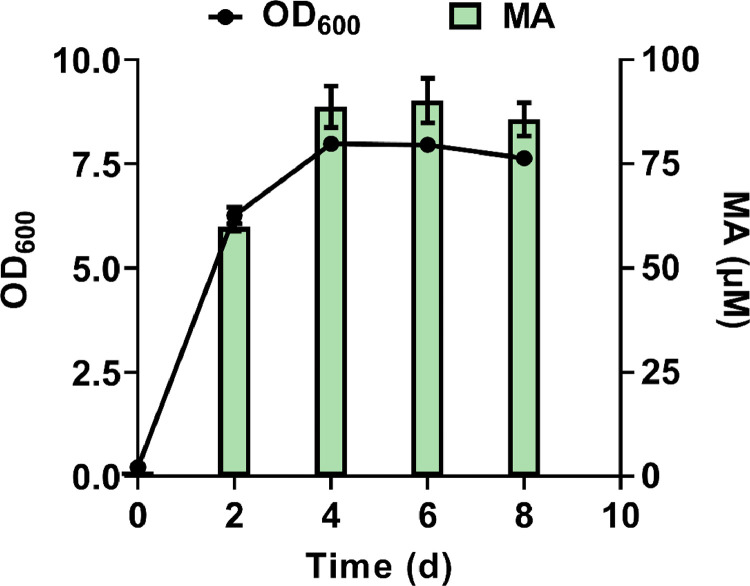
Cell growth and muconic acid accumulation during the co-cultivation of *Raoultella* sp. DY2415 and *P. putida* KT2440-ΔRBC. Herein, 25 mg pre-treated polystyrene films were added to 50 mL of the co-cultivation system and incubated at 30 °C, 220 rpm. Three parallel experiments were carried out.

To further evaluate the biodegradation of the PS films during co-cultivation, MA levels were measured every two days (Fig. 5). After two days of incubation, MA accumulated to a concentration of 59.9 μM. As the biomass increased, MA continued to accumulate, reaching a concentration of 88.73 μM on the fourth day. After this time, biomass and MA accumulation began to stabilize (Fig. 5). Simultaneously, we measured MA production over 24 h using different weights of pre-treated PS films. The results showed a 77.17% increase in MA accumulation upon the addition of 50 mg of pre-treated PS film compared to that with 30 mg. These results further suggest that MA was synthesized from the BA substrate produced upon PS degradation.

**Fig. 6:**
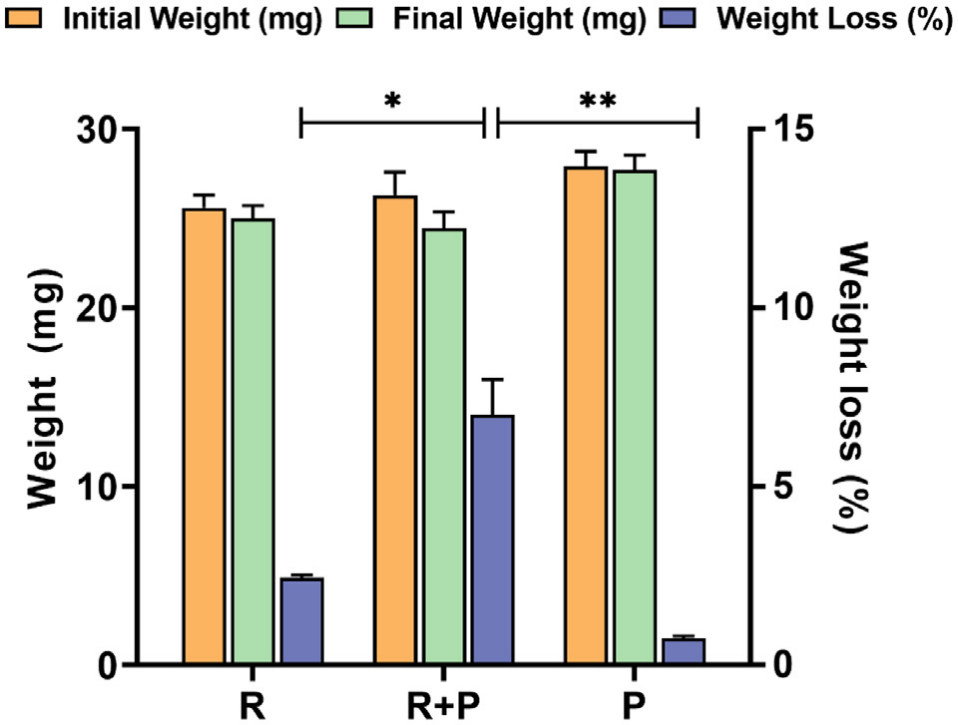
The weight changes of the pre-treated polystyrene films after *Raoultella* sp. DY2415 individual cultivation (R), *Raoultella* sp. DY2415 and *P. putida* KT2440-ΔRBC co-cultivation (R+P), and *P. putida* KT2440-ΔRBC individual cultivation (P) for one week. The inoculation ratio of the two bacteria during co-cultivation is 1:1. Three parallel experiments were carried out for each sample. Statistical analysis was conducted using Student's *t*-test. Asterisks (*) indicate statistical significance (0.05 < ns < 1, *p < 0.05, **p < 0.01, ***p < 0.001, ****p < 0.0001).

We also observed weight loss in the pre-treated PS films after one week of incubation with the investigated microorganisms. The pre-treated PS film incubated with *P. putida* KT2440-ΔRBC only exhibited a weight loss of 0.75 ± 0.07%, whereas the pre-treated PS film incubated with *Raoultella* sp. DY2415 only exhibited a weight loss of 2.45 ± 0.07% (Fig. 6). This weight loss is slightly higher than that previously reported for PS degradation by *Raoultella* sp. DY2415, suggesting that UV irradiation enhances PS biodegradation [28]. More importantly, the pre-treated PS films incubated with both *Raoultella* sp. DY2415 and *P. putida* KT2440-ΔRBC exhibited a weight loss of 7.0 ± 0.99%, 2.85 times higher than that of pure *Raoultella* sp. DY2415 (Fig. 6). In this study, the observed 7.0 ± 0.99% weight loss in the pre-treated PS films after one week of incubation represents the highest degradation rate measured across the experimental conditions. *Enterococcus* sp. isolated from wax moth larvae showed only 3.4% weight loss in response to PS for one week [42]. Most documented PS-degrading microorganisms achieve only moderate weight loss (1–10%) after prolonged incubation (1–2 months) [11,18,34]. This result indicates that the co-cultivation of *Raoultella* sp. DY2415 and *P. putida* KT2440-ΔRBC could more efficiently degrade pre-treated PS films compared to cultivation with *Raoultella* sp. DY2415 alone. In 2023, Li et al. found that the PS degradation efficiency of XP1 and XP2 bacteria under co-culture was 2.6 times higher than that under bacterial treatment with either XP1 or XP2, indicating the existence of interspecific assistance or the mutual use of metabolites between the two bacteria [44]. The authors believe that interspecific cooperation and competition are the key factors affecting the degradation efficiency of plastics by a bacterial co-culture. In this study, the co-cultivation of *Raoultella* sp. DY2415 and *P. putida* KT2440-ΔRBC could more efficiently degrade pre-treated PS films than cultivation with *Raoultella* sp. DY2415 alone, indicating that there may be a synergistic effect between the two bacteria that is beneficial for degradation.

**Fig. 7:**
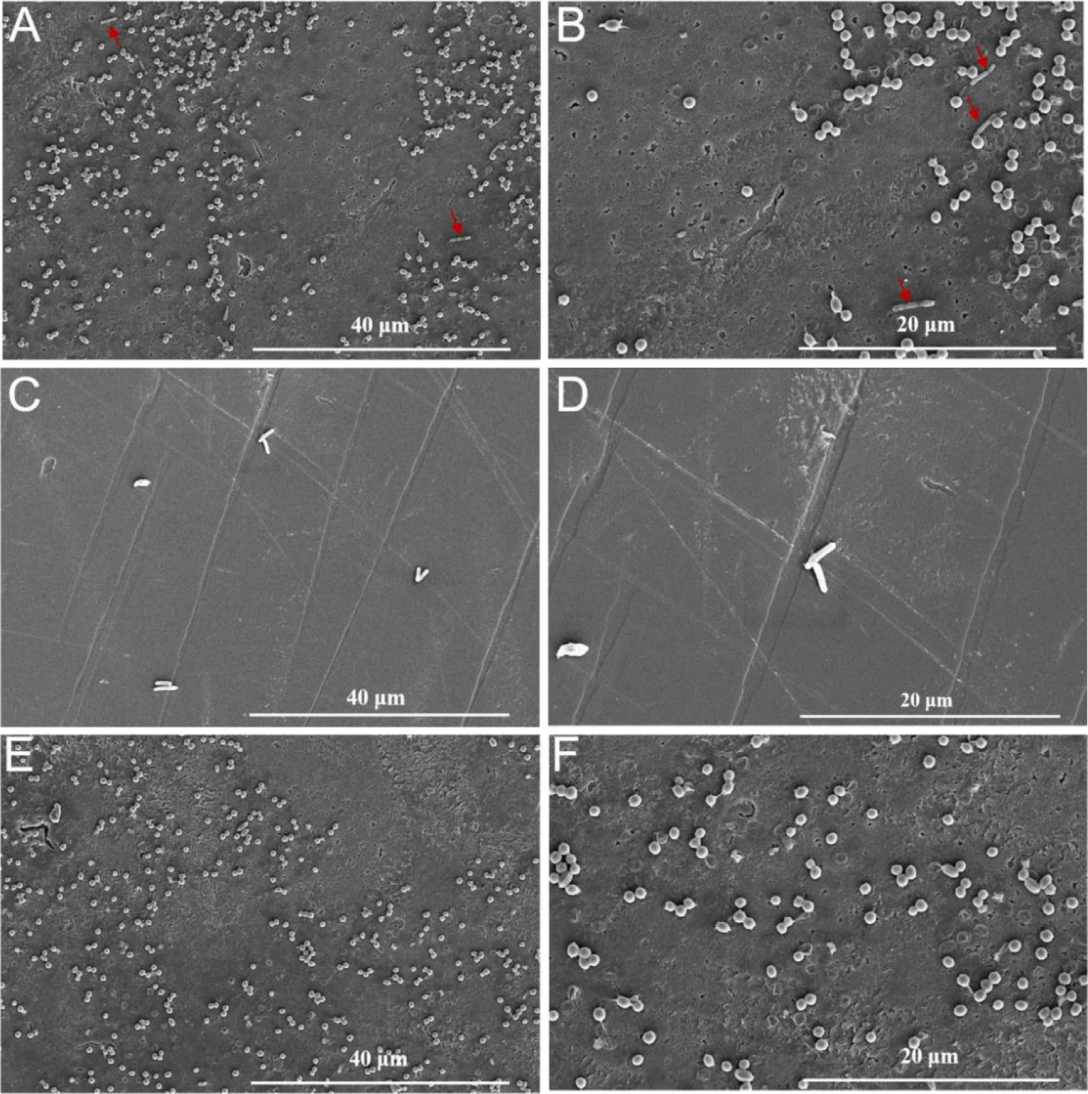
Scanning electron microscopy of the biodegradation of the pre-treated polystyrene (PS) films. Shown are the PS films after one week of co-cultivation with *Raoultella* sp. DY2415 and *P. putida* KT2440-ΔRBC. Magnification 5,000× (A) and 10,000× (B). The red arrow represents *P. putida* KT2440-ΔRBC. The PS films after one week of cultivation with *P. putida* KT2440-ΔRBC, magnification 5,000× (C) and 10,000× (D). The PS films after one week cultivation with *Raoultella* sp. DY2415, magnification 5,000× (E) and 10,000× (F).

Biodegradation of the PS films was characterized using SEM. As shown in Fig. 7A, the two types of microorganisms clearly attached to the surface of the pre-treated PS film. The spherical bacteria were *Raoultella* sp. DY2415 and the rod-shaped bacteria were *P. putida* KT2440-ΔRBC. Among these, *Raoultella* sp. DY2415 accounts for majority of the attached bacteria, while *P. putida* KT2440-ΔRBC only accounted for a small portion. This phenomenon is similar to previous studies, indicating that the first step in plastic degradation is the attachment of bacteria to the surface of the plastic substrate [45]. The PS films incubated with individual cultures of *Raoultella* sp. DY2415 exhibited some small pits and holes (Fig. 7). However, the PS films incubated with individual *P. putida* KT2440-ΔRBC showed only a small amount of bacterial colonization. In this setup, the morphology of the PS film was smooth without pits and holes, indicating that individual *P. putida* KT2440-ΔRBC cannot biodegrade the PS films.

**Fig. 8:**
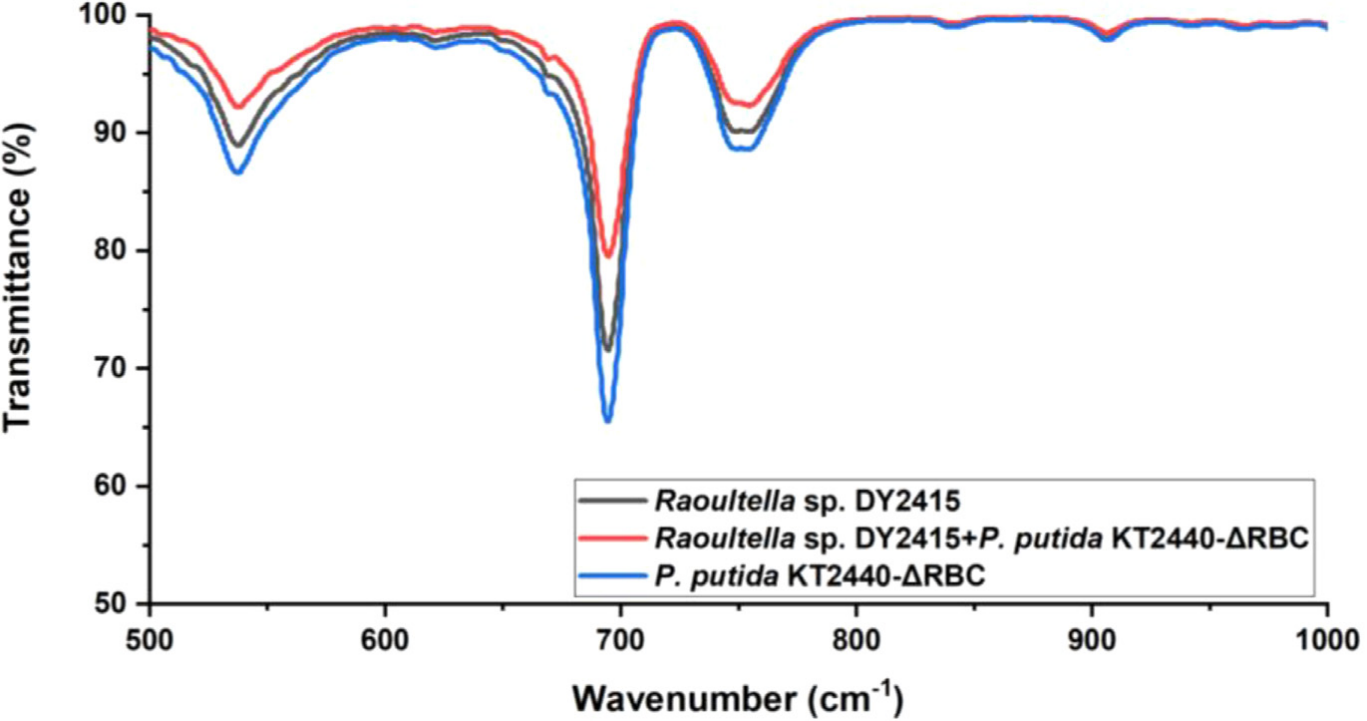
Fourier transform infrared analysis of the pre-treated polystyrene films after one week incubation.

To further characterize the biodegradation effect of the bacterial co-culture, we performed Fourier transform infrared (FTIR) spectrometry (Fig. 8). Compared to cultivation with *P. putida* KT2440-ΔRBC alone, which cannot biodegrade PS films, the vibration peaks at 696 cm^−1^ and 755 cm^−1^ disappeared in both the *Raoultella* sp. DY2415 monoculture and the co-culture of *Raoultella* sp. DY2415 and *P. putida* KT2440-ΔRBC. This reduction corresponds to C-H stretching vibrations; the peak abrogation was more pronounced in the co-culture group than in the corresponding monocultures. The disappearance of the vibration peaks at 696 cm^−1^ and 755 cm^−1^ indicated that the benzene ring dissociated after biodegradation [46,47]. The FTIR results were consistent with the previous weight loss and SEM results, indicating that co-cultivation can improve the degradation efficiency of PS.

The efficiency of pollutant biodegradation by bacterial consortia depends on intricate metabolic cross-feeding and exchange networks among community members, which serve as fundamental prerequisites for substrate mineralization [48]. In 2021, Zhang et al. reported enhanced degradation of PAHs by mixed cultures of *Mycobacterium* sp. strain A1-PYR and *Sphingomonas* sp. strain PheB4, compared to their respective pure cultures. Moreover, *Sphingomonas* sp. PheB4 utilizes 4-phenanthrol produced by *Mycobacterium* sp. A1-PYR. This interaction accelerates pyrene degradation by removing inhibitory metabolites [27]. In this study, the co-cultivation of *Raoultella* sp. DY2415 and *P. putida* KT2440-ΔRBC enhanced the degradation of PS, while *P. putida* KT2440-ΔRBC cannot degrade PS. The genome of *Raoultella* sp. DY2415 revealed diverse metabolic pathways for aromatic compounds, including BA, chlorocyclohexane, chlorobenzene, aminobenzoate, and fluorobenzoate. Previous experimental results have indicated that *Raoultella* sp. DY2415 possessed a novel BA metabolic pathway that differed from that of *P. putida* KT2440 [38]. The two-strain co-culture system employed in this study exhibited significantly greater metabolic complexity than their respective monocultures. Because these pathways sometimes differ for the same compounds, future experiments are needed to understand how this system improves the PS degradation efficiency.

## 4 Conclusion

In this study, we verified the degradation of PS and the biosynthesis of MA with the co-cultivation of *Raoultella* sp. DY2415 and *P. putida* KT2440-ΔRBC. This co-cultivation system successfully converted PS waste into the high-value chemical MA. As proof of this concept, our study demonstrated the feasibility of PS biodegradation and upcycling via microbial co-cultivation. This further confirms the capability of *Raoultella* sp. DY2415 to biodegrade PS waste and provides a novel approach for the management of other recalcitrant environmental pollutants.

## Data Availability Statement

The data supporting the findings of this study are included in the published article as well as in the Supplementary Materials available online.

## CRediT authorship contribution statement

**Yingbo Yuan:** Writing – original draft, Resources, Methodology, Investigation, Conceptualization. **Tianyuan Su:** Writing – review & editing, Writing – original draft, Validation, Funding acquisition. **Yi Zheng:** Methodology. **Baoyue Liu:** Methodology. **Yuanfei Han:** Methodology. **Zhongcan Wang:** Methodology. **Quanfeng Liang:** Writing – review & editing, Resources, Funding acquisition, Conceptualization. **Longyang Dian:** Writing – review & editing, Funding acquisition. **Qingsheng Qi:** Writing – review & editing, Validation, Resources, Conceptualization.

## Declaration of Competing Interest

The authors declare that they have no known competing financial interests or personal relationships that could have appeared to influence the work reported in this paper.

## Appendix Supplementary materials

Image, application 1
