# Supplementary material for: Bacterial co-cultivation for the degradation of polystyrene plastics
Source: Eng Microbiol. 2025 Aug 19;5(4):100232. doi: 10.1016/j.engmic.2025.100232 (PMC12967824; doi:10.1016/j.engmic.2025.100232)
Supplement: Supplementary file 1 [file mmc1.docx]

**Supplementary Materials**

**Bacterial co-cultivation for the degradation of polystyrene plastics**

Yingbo Yuan^#^, Tianyuan Su^#^, Yi Zheng, Baoyue Liu, Yuanfei Han, Zhongcan Wang, Quanfeng Liang, Longyang Dian*, Qingsheng Qi*

State Key Laboratory of Microbial Technology, Shandong University, Qingdao, 266237, People’s Republic of China

# These authors contributed equally to this work.

*** Correspondence：**

Longyang Dian

Email: longyang_dian@sdu.edu.cn

Qingsheng Qi

Email: qiqingsheng@sdu.edu.cn

Tel: +86-532-58632580

**Table S1 Strains and plasmids used in this study**

| **Strains** | **Description** | **Source** |
| --- | --- | --- |
| *E. coli* DH5α | *F^–^ endA1 glnV44 thi-1 recA1 relA1 gyrA96 deoR nupG purB20 φ80dlacZ∆M15 ∆(lacZYA-argF) U169, hsdR17(r_K_^–^m_K_^+^), λ^–^* | Lab stock |
| *Raoultella* sp. DY2415 | Isolated and identified from petroleum-contaminated soil located in Shandong which can degrade PE and PS films. | Lab stock |
| *P. putida* KT2440 | Wile-type strain | Lab stock |
| *P. putida* KT2440-ΔRBC | KT2440 derivative with the deletion of *catRBC* | This study |
| pK18mobSacB | Allelic exchange vector, *oriColE1 Mob+, lacZα, sacB, Kan^R^* | Lab stock |
| pK18-*ΔcatRBC* | pK18mobSacB for deletion of *catRBC*, *Kan^R^* | This study |

**Table S2 Primers used in this study**

| **Primers** | **Sequences (5’-3’)** | **Purposes** |
| --- | --- | --- |
| pK18-FP | GGATCCTCTAGAGTCGACCT | pK18mobsacB |
| pK18-RP | AATTGCGTTGCGCTCACTGC | pK18mobsacB |
| catABC-FP | GACCCTTTCTGCCTTGCC | *catRBC* |
| catABC-RP | GTTGATCTGCGTGGTCAGG | *catRBC* |
| catRBC-down-FP | GTGGGCATGGTGTGTTTCGCACCTGTATGCCTG | *catRBC* down arm |
| catRBC-down-RP | GACTCTAGAGGATCCCCCGTCGCGGGTGGCA | *catRBC* down arm |
| catRBC-up-FP | GCATACAGGTGCGAAACACACCATGCCCACAG | *catRBC* up arm |
| catRBC-up-RP | GAGCGCAACGCAATTTCGATGATGATCAGCCTTGAG | *catRBC* up arm |

**Table S3. Benzoate metabolism gene in *Raoultella* sp. DY2415.**

| Gene Number | Gene Annotation |
| --- | --- |
| 0946 | putative acetyl-CoA acetyltransferase |
| 1460 | 3-ketoacyl-CoA thiolase |
| 1461 | Fatty acid oxidation complex subunit alpha |
| 2163 | 2-keto-4-pentenoate hydratase |
| 2164 | Acetaldehyde dehydrogenase |
| 2165 | 4-hydroxy-2-oxovalerate aldolase |
| 2232 | hypothetical protein |
| 2233 | Acetyl-CoA acetyltransferase |
| 2388 | 3-oxoadipate CoA-transferase subunit A |
| 2389 | 3-oxoadipate CoA-transferase subunit B |
| 2390 | Beta-ketoadipyl-CoA thiolase |
| 2391 | 3-carboxy-cis, cis-muconate cycloisomerase |
| 2392 | 3-oxoadipate enol-lactonase 1 |
| 2393 | 4-carboxymuconolactone decarboxylase |
| 2735 | 1,6-dihydroxycyclohexa-2,4-diene-1-carboxylate dehydrogenase |
| 2736 | Toluate 1,2-dioxygenase electron transfer component |
| 2737 | Toluate 1,2-dioxygenase subunit beta |
| 2738 | Toluate 1,2-dioxygenase subunit alpha |
| 2739 | Catechol 1,2-dioxygenase |
| 2740 | Muconolactone Delta-isomerase |
| 2741 | Muconate cycloisomerase 1 |
| 2753 | hypothetical protein |
| 2770 | Tautomerase PptA |
| 2783 | hypothetical protein |
| 2846 | Protocatechuate 3,4-dioxygenase beta chain |
| 2847 | Protocatechuate 3,4-dioxygenase alpha chain |
| 2894 | 3-hydroxyadipyl-CoA dehydrogenase |
| 2896 | 2,3-dehydroadipyl-CoA hydratase |
| 2968 | hypothetical protein |
| 2969 | hypothetical protein |
| 3000 | hypothetical protein |
| 4618 | p-hydroxybenzoate hydroxylase |
| 4748 | hypothetical protein |
| 5104 | Fatty acid oxidation complex subunit alpha |
| 5105 | 3-ketoacyl-CoA thiolase |
| 5194 | putative 13.8 kDa protein in nqo9-nqo10 intergenic region |

**
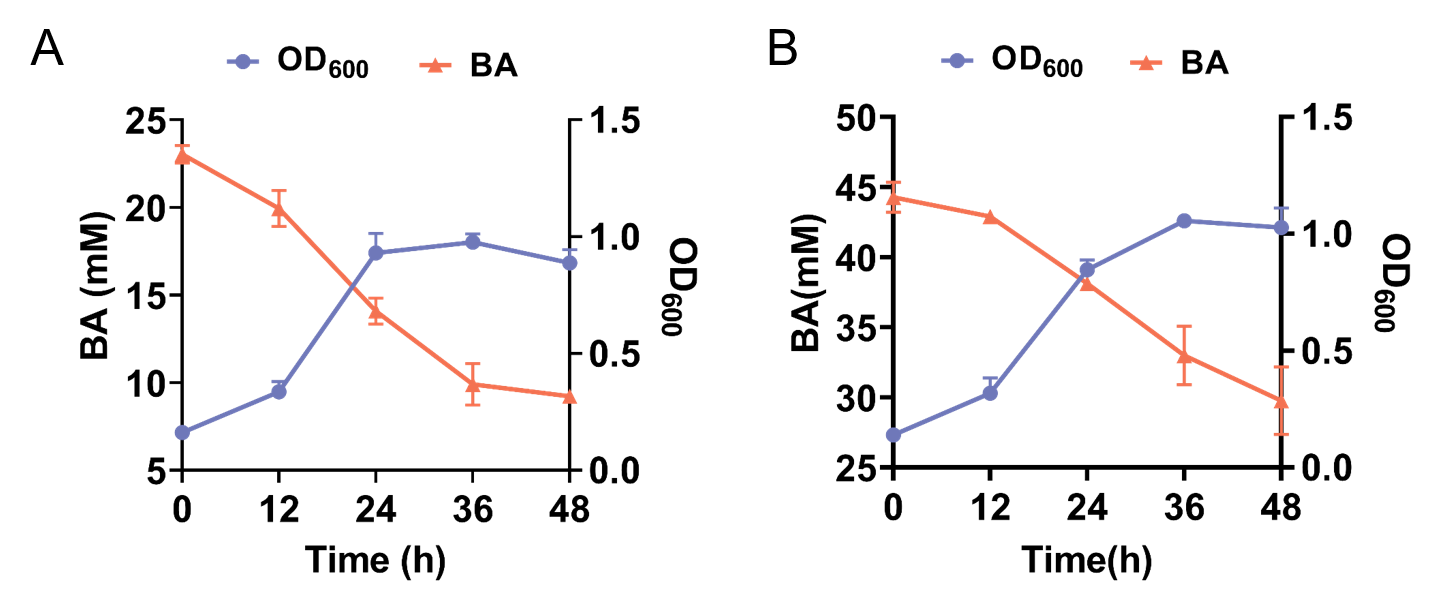
**

**Fig. S1 *Raoultella* sp. DY2415 metalize BA in LB medium supplied with 20 mM benzoate (A) and 40 mM benzoate (B). Each experimental group was conducted twice, the mean values (± standard deviation) were visualized.**

**
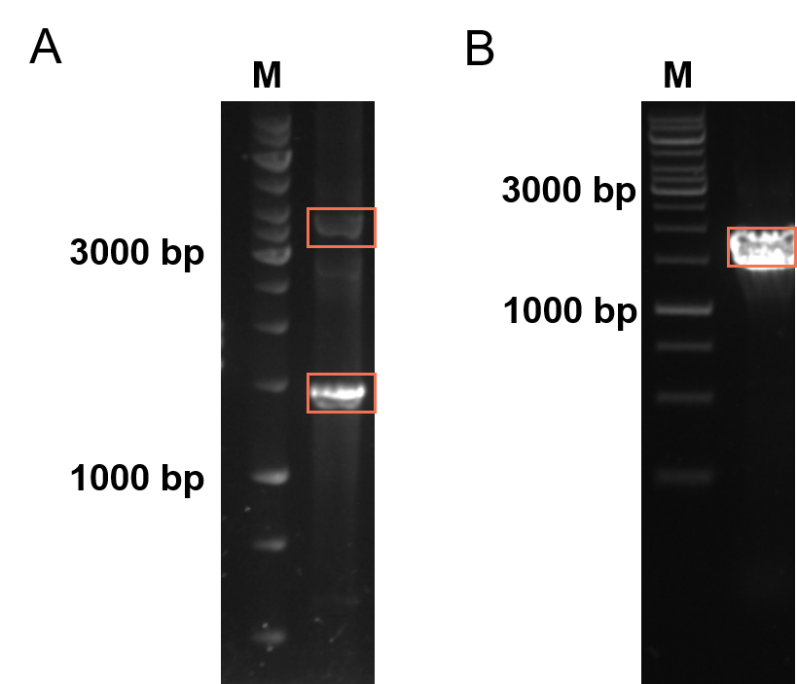
**

**Fig. S2 The agarose electrophoresis detection of DNA bands before (A) and after (B) knock out the MA metabolite and regulate gene *catB, catC, catR* in *P. putida* KT2440. (A) showed two DNA bands and the above band represented the homology arm sequences and the gene *catB, catC, catR,* and the below band represented the homology arm sequences*.***

**
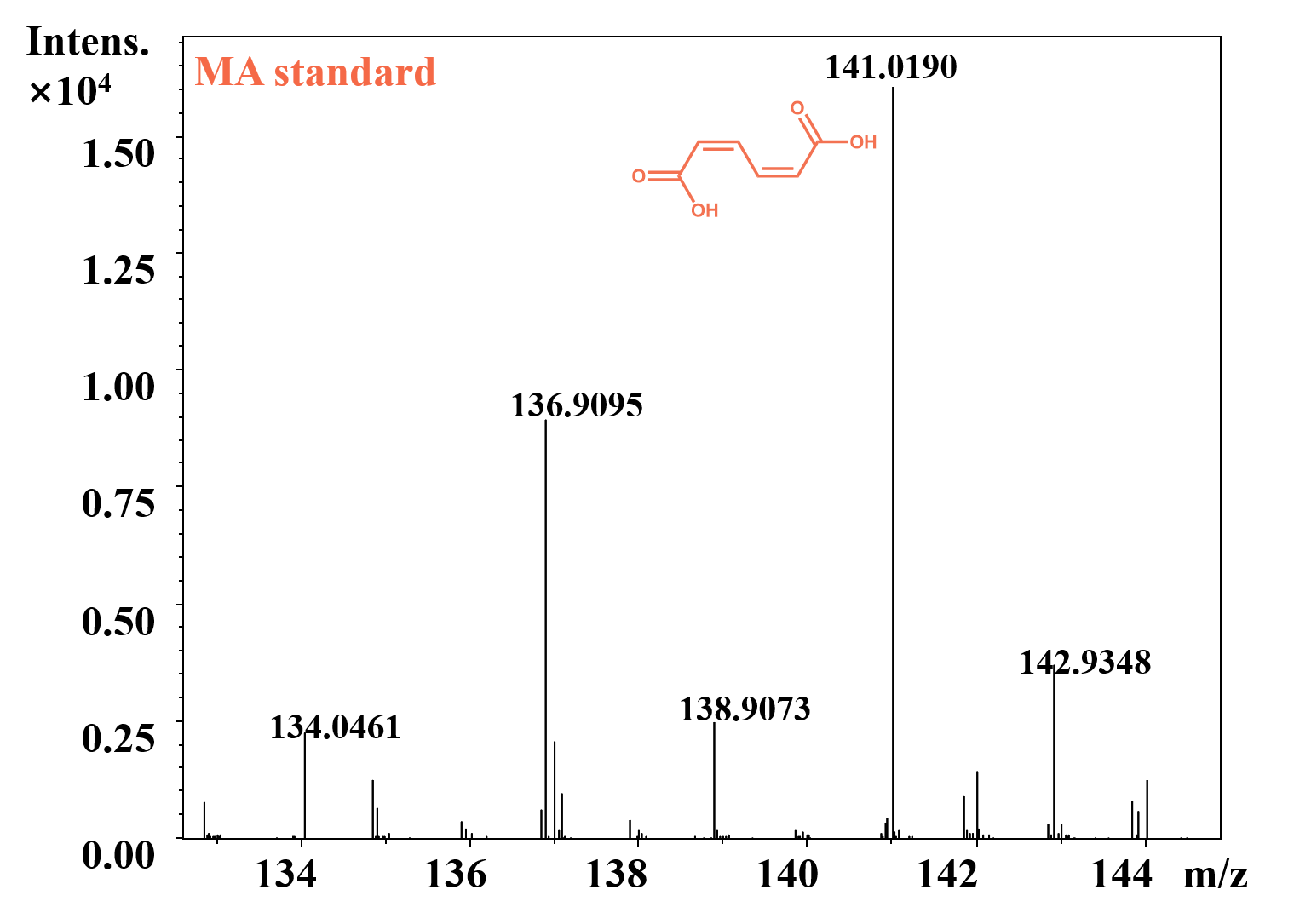
**

**Fig. S3 HPLC-MS spectrum of MA standard.**

**
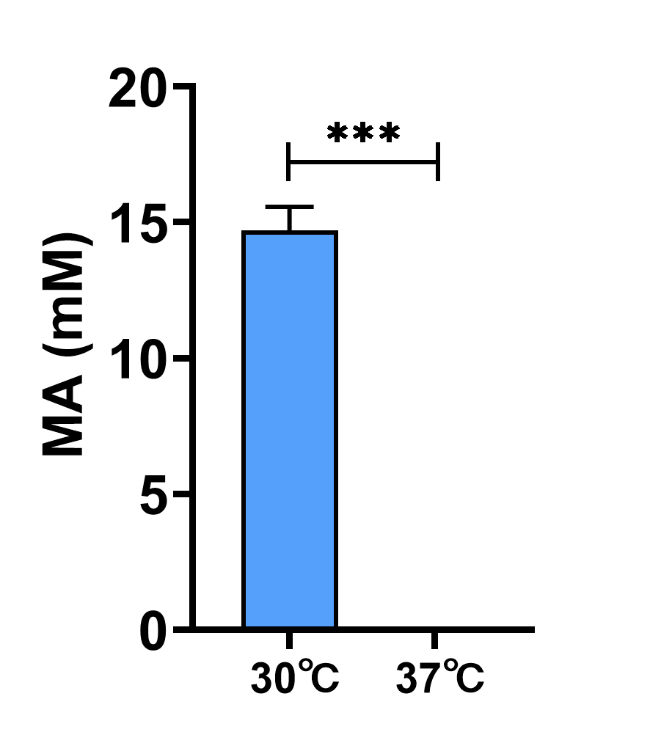
**

**Fig. S4 The MA accumulation of cocultivation at different temperature during 12 h. The data featured in panels A and B were produced by averaging the results obtained for 2 independent reactions. Error bars indicate standard deviations between the independent reactions. Statistical analysis was conducted using Student's t-test, and the asterisk (*) represents the statistical significance (0.05 < ns < 1, *p < 0.05, **p < 0.01, ***p < 0.001, ****p < 0.0001).**

**
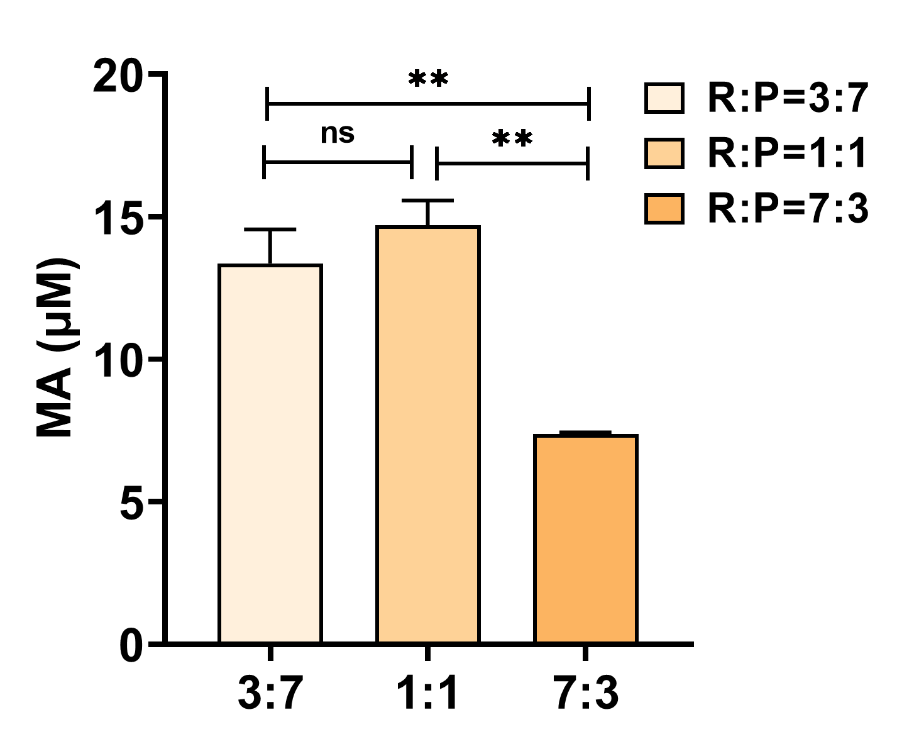
**

**Fig. S5 The MA accumulation of cocultivation at different inoculation ratios during 12 h. The data featured in panels A and B were produced by averaging the results obtained for 2 independent reactions. Error bars indicate standard deviations between the independent reactions. Statistical analysis was conducted using Student's t-test, and the asterisk (*) represents the statistical significance (0.05 < ns < 1, *p < 0.05, **p < 0.01, ***p < 0.001, ****p < 0.0001).**
